# GWAS‐assisted genomic selection for achieving high‐precision early sex prediction in *Populus deltoides*

**DOI:** 10.1002/tpg2.70175

**Published:** 2026-01-16

**Authors:** Xinglu Zhou, Min Zhang, Lei Zhang, Jianjun Hu

**Affiliations:** ^1^ State Key Laboratory of Tree Genetics and Breeding Research Institute of Forestry Chinese Academy of Forestry Beijing China; ^2^ Co‐Innovation Center for Sustainable Forestry in Southern China Nanjing Forestry University Nanjing China

## Abstract

Poplar (*Populus* spp.) breeding programs increasingly prioritize the development of male varieties due to the environmental issues caused by female catkins. Therefore, there is an urgent need for a reliable technique for early sex identification in *Populus*. Genomic selection (GS), as an efficient predictive method, offers a promising solution for early sex identification in poplar. In this study, we conducted genomic prediction for sex in *Populus deltoides*. Using five full‐sib families of *P. deltoides* as the reference population, we identified 801 sex‐associated loci through GWAS and precisely localized the sex determination region at the telomeric end of chromosome 19. We evaluated 14 GS statistical models using fivefold cross‐validation under six marker densities. The results showed significant differences in prediction accuracy (PA) among different statistical models, ranging from 0.19 to 0.79, with the gradient boosting decision tree exhibiting the highest accuracy and stability. Notably, single nucleotide polymorphisms selected through GWAS significantly improved PA compared to random markers, achieving a corrected accuracy of 0.999. Using the optimal model and markers, we predicted the sex of 505 progenies from 27 full‐sib families, with over 90% of the predictions being accurate. Overall, this study achieved high‐accuracy sex prediction in *P. deltoides* through genome prediction, providing a novel and efficient method for poplar sex identification.

## Core Ideas

- Genomic selection offers a novel approach for early sex identification in *Populus deltoides*.
- The gradient boosting decision tree (GBDT) model demonstrated the best overall performance in sex prediction for *Populus deltoides*.
- Genome‐wide association study (GWAS)‐assisted genomic selection significantly enhances the accuracy of sex prediction.
- The optimal prediction model achieved an accuracy of over 90% in sex prediction for *Populus deltoides*.

## Plain Language Summary

Poplar breeding programs increasingly prioritize the development of male varieties due to environmental issues caused by female catkins. Current sex identification methods are inefficient, highlighting the urgent need for a reliable technique for early sex identification in *Populus deltoides*. Using a multi‐generational hybrid population of *P. deltoides*, we explored the potential of genomic selection (GS) for poplar sex prediction. The evaluation results of the GS statistical models showed significant differences in sex prediction accuracy (PA) among different models, with gradient boosting decision tree having the best overall performance. Notably, GWAS‐selected single nucleotide polymorphisms significantly improved PA compared to random markers. Model accuracy remained stable at high marker densities but decreased significantly at lower densities. This study demonstrates the feasibility of high‐accuracy sex prediction in *P. deltoides* through GS, providing a novel and efficient method for early sex identification in poplar breeding programs.

**Abbreviations**
DBH: diameter at breast height
EN: elastic net regression
GBDT: gradient boosting decision tree
GEBV: genomic estimated breeding value
GS: genomic selection
GWAS: genome‐wide association study
LD: linkage disequilibrium
KRR: kernel ridge regression
PA: prediction accuracy
RF: random forest
SDR: sex determination region
SLR: sex‐linked region
SNP: single nucleotide polymorphism
VP: validation population
XGBoost: eXtreme gradient boosting

## 1 INTRODUCTION

Poplar (*Populus* spp.), a woody plant of significant ecological and economic value, is widely used in shelterbelts, timber plantations, and ecological restoration (Taylor, 2002). Sex in poplar is closely associated with radial growth and ecological environment. From April to May each year, mature female trees produce large amounts of seed fluff, which can absorb airborne pollutants and other pollen allergens, potentially causing negative impacts on human activities and the ecological environment (Ortega et al., 2023; Pankova et al., 2016). Furthermore, studies have shown that male and female poplar trees exhibit differences in resistance to environmental stresses such as drought, salt stress, and soil salinization (He et al., 2022; Yu et al., 2023). Consequently, poplar breeding programs increasingly prioritize the development of male varieties. However, poplar has a long juvenile phase, typically requiring 6–7 years to reach the flowering stage, making it difficult to determine its sex before this period. Understanding the mechanisms of sex determination and achieving early sex identification have significant importance and practical value for the genetic improvement of poplar, particularly in hybrid breeding programs.

Similar to other dioecious plants, poplars possess a complex sex determination system (Cronk, 2005; Müller et al., 2020). Recent research has revealed that at least two sex determination systems, ZW and XY, exist within the genus *Populus*. An interesting finding is that Li et al. (2024) identified and confirmed a novel ZY sex genotype through both natural and artificial hybrid populations, which represents a crossover between the XY and ZW systems. Distinct sex determination regions (SDRs) have been identified even among species with the same system (Gladysh et al., 2024). For example, in the XY system, the SDR of *Populus euphratica* is located on chromosome 14 (Yang et al., 2021), while that of *Populus deltoides* resides at the distal end of chromosome 19 (Xue et al., 2020). The SDR in poplars is highly conserved due to recombination suppression during evolution, which prevents genetic exchange and facilitates the precise identification of SDRs (Rifkin et al., 2020).

Currently, early sex identification in poplar primarily relies on molecular marker technology, which has been applied to sex identification in *P. deltoides*, *Populus simonii*, *Populus davidiana*, *Populus tomentosa*, and *Populus tremula* (Gladysh et al., 2024; Leite Montalvão et al., 2022; Wang et al., 2023). However, due to the specificity of SDR regions among different poplar species, these primers are often not universally applicable. In practical applications, species‐specific primers must be designed, which is time‐consuming and labor‐intensive for population‐level studies. Genomic selection (GS), as an advanced breeding strategy, is gradually revolutionizing the genetic improvement of poplars (Ahmar et al., 2021; Alemu et al., 2024), while also providing a novel approach for early sex identification in poplars.

The accuracy of genomic prediction is closely related to factors such as statistical models, marker density, and the genetic relationships within the population (Xu et al., 2020). Although numerous statistical models have been developed, no single model has proven universally suitable for most GS studies (Heslot et al., 2012). For qualitative traits like poplar sex, which are controlled by relatively few genes, an excessive number of markers can lead to model overfitting and negatively impact prediction accuracy (Sallam et al., 2015). Therefore, selecting markers that are closely associated with the trait is critical for improving prediction accuracy. Genome‐wide association study (GWAS), as a powerful association tool, can accurately identify sex‐related markers (Cortes et al., 2021). The integration of GWAS with GS for genomic prediction has been preliminarily demonstrated in species such as spruce, pear, and peach (Bernhardsson et al., 2021; Li et al., 2020; Li et al., 2023; Minamikawa et al., 2018). In addition to assisting genomic selection breeding, GWAS also provides robust evidence for unraveling the genetic mechanisms underlying sex determination in poplars.

*Populus deltoides* is native to North America and shows fast growth, high‐quality wood, and strong resistance. Since its introduction to China from the 1970s (Wang & Kung, 1987), it has been highly valued and widely used in hybrid breeding programs (Dobhal et al., 2023). Based on multi‐generation hybridization, our research team established a multi‐generation hybrid population of *P. deltoides* and performed whole‐genome re‐sequencing. Using sex and genotype data from the reference population, we employed GWAS to identify key marker loci and potential genes associated with sex determination in *P. deltoides*. We then evaluated and determined the most suitable sex statistical model, optimized the genomic selection markers, and established an efficient sex prediction model for *P. deltoides*, achieving precise sex prediction in breeding populations. The study aims to explore the feasibility of early sex prediction in hybrid populations through the integration of GWAS and GS, providing sex information for subsequent genomic selection and offering a new approach for early sex identification in poplars.

## 2 MATERIALS AND METHODS

### 2.1 Materials and phenotypic data collection

The plant materials consisted of 765 hybrid progenies from 32 multi‐generational full‐sib families of *Populus deltoides*. Among them, 260 large mature trees from five full‐sibling families constituted the reference population. These trees were planted in 2014 at the Daxing Forest Farm in Beijing. The maternal parent of five full‐sibling families was *P. deltoides* “Danhong” (DHY), while the paternal parent was elite clone of *P. deltoides* (Table 1). The breeding population, comprising 505 trees, was randomly sampled at a proportion of 20% from 27 full‐sib families. The parents of these families were carefully selected based on the genetic background of the reference population parents to ensure that both the reference and breeding populations share a similar genetic structure.

**TABLE 1:** Genetic background of the hybrid parental lines in the reference population.

Name	Sex	Latin	Maternal parent	Paternal parent
DHY	Female	*P. deltoides* “Danhong”	*P. deltoides* “55/65”	*P. deltoides* “2KEN8”
3_54	Male	*P. deltoides* “Zhongcheng4”	*P. deltoides* “Danhong”	*P. deltoides* “Chuangxin”
16_27	Male	*P. deltoides* “Beikang”	*P. deltoides* “Danhong”	*P. deltoides* “D175”
17_2	Male	*P. deltoides* “Chuangxin”	*P. deltoides* “Nankang 1A”	*P. deltoides* “Imperial”
276	Male	*P. deltoides* “Nanyang”	*P. deltoides* “55/65”	*P. deltoides* “2KEN8”
1_278	Male	*P. deltoides* “Zhongcheng2”	*P. deltoides* “Danhong”	*P. deltoides* “Nanyang”

For the reference population, the sex of each plant was initially determined in mid‐April 2022, after the male flowers had swelled. In early June of the same year, the sex was verified again based on the seed fluff from female flowers, with females recorded as 1 and males as 2. In 2023, the sex of each tree was re‐checked to ensure accuracy, and the height (H) and diameter at breast height (DBH) of each tree were also measured. All materials were propagated asexually in the nursery of the Research Institute of Forestry, Chinese Academy of Forestry.

**Core Ideas**

- Genomic selection offers a novel approach for early sex identification in *Populus deltoides*.
- The gradient boosting decision tree (GBDT) model demonstrated the best overall performance in sex prediction for *Populus deltoides*.
- Genome‐wide association study (GWAS)‐assisted genomic selection significantly enhances the accuracy of sex prediction.
- The optimal prediction model achieved an accuracy of over 90% in sex prediction for *Populus deltoides*.

### 2.2 Genotypic data and genotyping

The whole‐genome re‐sequencing data were derived from our previous study (Zhou et al., 2025) and have been deposited in the National Genomics Data Center (https://ngdc.cncb.ac.cn/?lang=en) under BioProject accession number PRJCA033212. Briefly, fresh leaf tissues were collected from each tree in both the reference and breeding populations for DNA extraction and subsequent whole‐genome resequencing. The *P. deltoides* “Danhong” genome, which achieves telomere‐to‐telomere level assembly for most chromosomes, including chromosome 19, was used as the reference genome (BioProject: PRJCA033198). Variant detection was performed using GATK v4.2.5.0 (McKenna et al., 2010). Strict quality control was applied using VCFtools v0.1.16 (Danecek et al., 2011) for subsequent GWAS analysis, with removing single nucleotide polymorphism (SNP) missing rate <0.02, quality score ≤30, minor allele frequency <0.05, minimum read depth <3, and exclusion of SNPs significantly deviating from the Hardy–Weinberg equilibrium. After quality filtering, approximately 1.14 million high‐quality SNP markers were remained, which were imputed using BEAGLE (Browning et al., 2018) for subsequent GS analyses.

### 2.3 Genome‐wide association analysis and candidate gene identification

GWAS was conducted to identify sex‐linked regions (SLRs) using the genotype and sex information of the reference population. The analysis was performed using the mixed linear model implemented in GEMMA v0.98.1 (Zhou & Stephens, 2012). To account for population structure and relatedness, the kinship matrix and principal component analysis were calculated using the Tassel software and incorporated as covariates in the model. The significance threshold for association was determined using the Bonferroni correction, set at *p* < 0.05/N, where N represents the total number of markers. Based on the population's linkage disequilibrium (LD) decay, 16 kb upstream and downstream of the significant loci were considered candidate regions. Candidate genes within these regions were identified using Bedtools (Quinlan & Hall, 2010), based on the genome annotation of *P. deltoides* “Danhong.”

### 2.4 Evaluation of genomic selection statistical models

To determine the optimal GS model for sex predicting, we evaluated the predictive performance of 14 widely used statistical models (Alemu et al., 2024; Zhou et al., 2025). These models were classified into four categories (Table 2): BLUP models (genomic best linear unbiased prediction and ridge regression best linear unbiased prediction; Endelman, 2011), Bayesian models (Bayesian A, Bayesian B, Bayesian C, Bayesian LASSO, and Bayesian ridge regression; Pérez & de los Campos, 2014), machine learning models (gradient boosting decision tree, random forest [RF], eXtreme gradient boosting [XGBoost], elastic net regression [EN], kernel ridge regression [KRR], and multiple kernel ridge regression based on reproducing kernel Hilbert space; Xu et al., 2020), and deep learning model (DNNGP; Wang et al., 2023). The predictive performance of each model was evaluated using a five‐fold cross‐validation approach. To avoid overfitting caused by an excessive number of neutral markers, we used vcflib v1.0.2 (Garrison et al., 2022) to randomly generate six high‐density marker subsets (N, 0.5N, 0.2N, 0.1N, 0.05N, and 0.01N), N refers to the total number of SNPs. The number of markers in these subsets ranged from 11,480 to 1,148,074. The statistical model demonstrating the best predictive performance across different marker densities was selected for subsequent analysis.

**TABLE 2:** The 14 genomic selection statistical models used in this study.

Model category	Model name	Abbreviation	Evaluation method	Running environment
BLUP class	Genomic best linear unbiased prediction	GBLUP	5‐Fold CV	R package “sommer”
	Ridge regression best linear unbiased prediction	RRBLUP	5‐Fold CV	R package “sommer”
Bayes class	Bayesian A	BayesA	5‐Fold CV	R package “BGLR”
	Bayesian B	BayesB	5‐Fold CV	R package “BGLR”
	Bayesian C	BayesC	5‐Fold CV	R package “BGLR”
	Bayesian LASSO	BL	5‐Fold CV	R package “BGLR”
	Bayesian ridge regression	BRR	5‐Fold CV	R package “BGLR”
Machine learning	Gradient boosting decision tree	GBDT	5‐Fold CV	Python3
	Random forest	RF	5‐Fold CV	Python3
	eXtreme Gradient Boosting	XGBoost	5‐Fold CV	Python3
	Elastic net regression	EN	5‐Fold CV	Python3
	Kernel ridge regression	KRR	5‐Fold CV	Python3
	Multiple kernel ridge regression based on reproducing kernel Hilbert space	MKRKHS	5‐Fold CV	Python3
Deep learning	Deep neural network Gaussian process	DNNGP	5‐Fold CV	DNNGP

### 2.5 Fivefold cross‐validation

Fivefold cross‐validation for model evaluation was conducted as follows: the reference population was randomly divided into five subsets, with four subsets serving as the training population for model training, and the remaining subset used as the validation populations (VPs). The average correlation coefficient between the true phenotypic values of the VPs and the genomic estimated breeding values (GEBVs) was used as the final evaluation metric. To reduce random error, all fivefold cross‐validation procedures were repeated 10 times.

### 2.6 GWAS‐assisted marker selection for GS

After determining the most suitable statistical model for sex prediction, we constructed seven marker subsets based on the GWAS association results: *p* ≤ 1, *p* ≤ 0.5, *p* ≤ 0.1, *p* ≤ 0.01, and *p* ≤ 0.001, significant marks, top100 marks, and top10 marks. Predictive accuracy was evaluated using the fivefold cross‐validation approach, repeated 10 times, with the average value as finally result. For each marker subset, an equal number of markers were randomly selected as a control group. Random markers were generated using vcflib v1.0.2 (Garrison et al., 2022). Based on the evaluation results, the optimal marker selection strategy for sex prediction was determined, and the influence of marker–trait associations on predictive accuracy was systematically explored.

### 2.7 Genomic prediction in the breeding population

Genotype data of the breeding population were extracted using VCFtools v0.1.16 (Danecek et al., 2011). Based on the established optimal sex prediction model, genomic prediction was performed to calculate the GEBV for each sample in the breeding population. For the prediction results, adjustments were made according to a 5% confidence interval. Specifically, GEBV values ranging from 0.95 and 1.05 were classified as female and recorded as 1, while those ranging from 1.95 and 2.05 were classified as male and recorded as 2.

## 3 RESULTS

### 3.1 Effect of sex differences on growth traits

**FIGURE 1:**
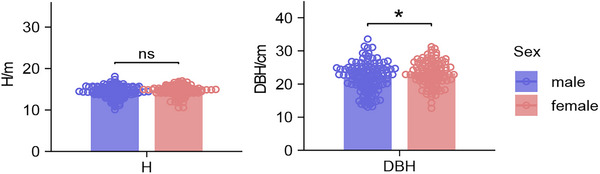
Statistical analysis of the 10‐year growth data of *P. deltoides* with different sexes. “ns” indicates no significant difference, and “*” indicates a significant difference at the 0.05 level. DBH, diameter at breast height; H, height.

Based on the sex identification results of the reference population during 2022–2023, we finally determined the sex of 235 trees, including 132 female and 103 male trees. The remaining 25 trees were excluded due to inconsistent results between the 2 years. A chi‐squared (*χ* ^2^) test revealed that the observed female‐to‐male ratio conformed to the Mendelian 1:1 segregation ratio, with a chi‐squared value of 3.59 (*χ* ^2^ = 3.84, *α* ≤ 0.05). At 10 years of age, the average height (H) of female trees was 14.37 m, while that of male trees was 14.63 m. The average DBH for female trees was 22.09 cm, compared to 23.15 cm for male trees. Statistical analysis showed no significant difference in tree height between female and male trees, but male trees exhibited a significantly larger DBH compared to female trees at *p* = 0.05 (Figure 1).

### 3.2 Identification of sex‐related markers

**FIGURE 2:**
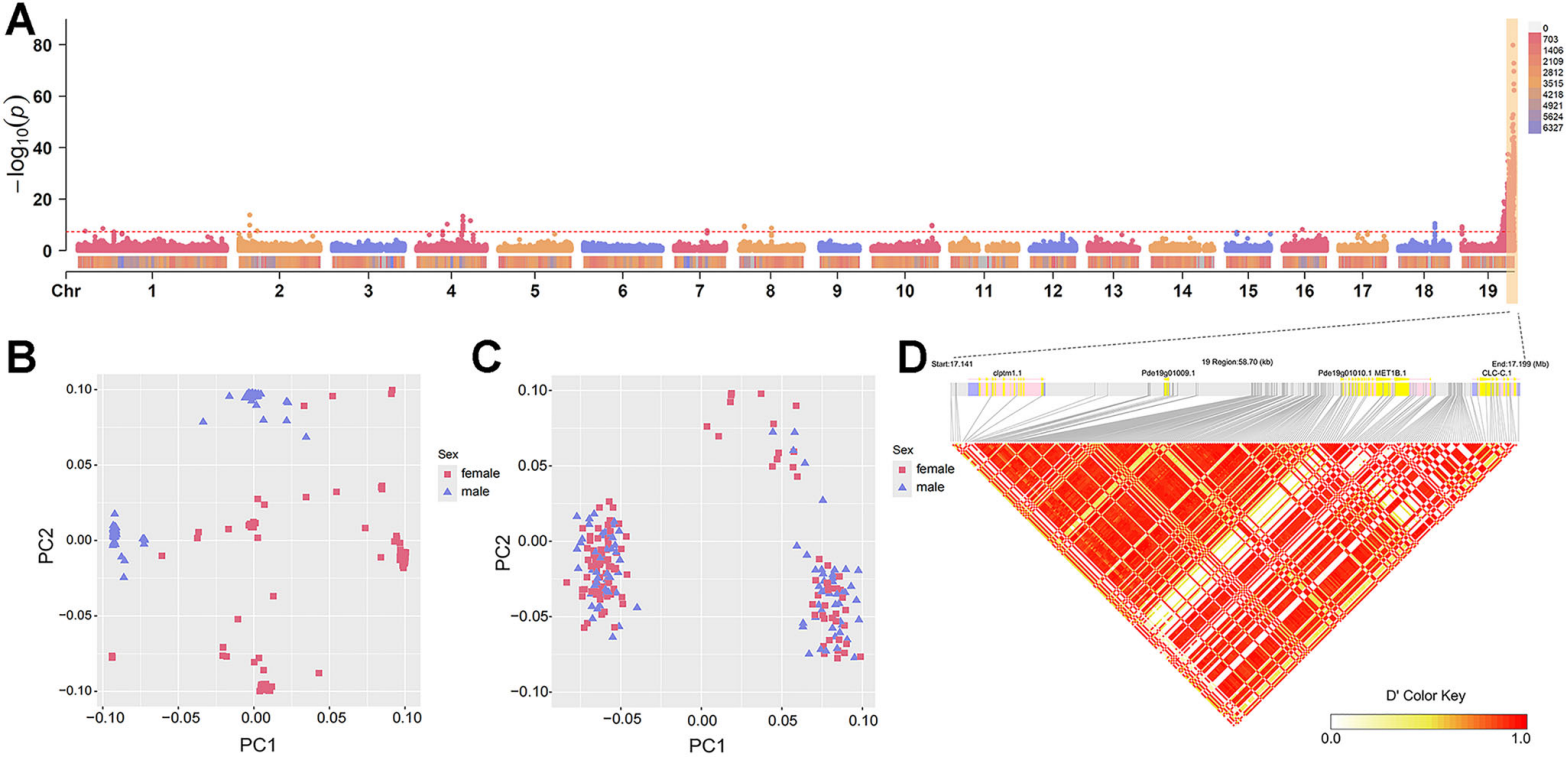
Sex genome‐wide association study (GWAS) and principal component analysis in *P. deltoides*. (A) Manhattan plot of the sex genome‐wide association analysis. (B) Principal component analysis results of significant markers. (C) Principal component analysis results of all markers. (D) Linkage disequilibrium (LD) heatmap of the sex determination region (SDR).

To identify SLRs across the genome, we conducted a GWAS using SNP markers from 235 trees. The Manhattan plot revealed a total of 801 SNPs surpassing the significance threshold (*p* ≤ 4.36×10^−7^), primarily located near the telomeric region of chromosome 19 (Figure 2A, Figure S1). This region aligns with the previously SDR of *P. deltoides*. The top two principal components of the loci within the SLR effectively separated the 235 trees into two groups corresponding to males and females (Figure 2B). In contrast, no clear separation was observed when using all markers (Figure 2C). These findings not only validate the accuracy of the GWAS results but also provide robust evidence for subsequent genomic prediction of sex.

### 3.3 LD analysis of the SDR

LD analysis near the peak markers revealed a high degree of linkage between markers in the 17.14‐17.20 Mb region of chromosome 19 (Figure 2D), consistent with the characteristics of the sex‐determining region. Using the DHY genome for annotation, five genes were identified within this region. Among these, *CLC‐C* and *MET1* have been previously reported to reside within the SDR of *P. deltoides*. Additionally, the region includes a highly conserved gene, *clptm1.1*, associated with cleft lip and palate transmembrane protein 1, which warrants further investigation.

### 3.4 The optimal GS statistical model for sex prediction

**FIGURE 3:**
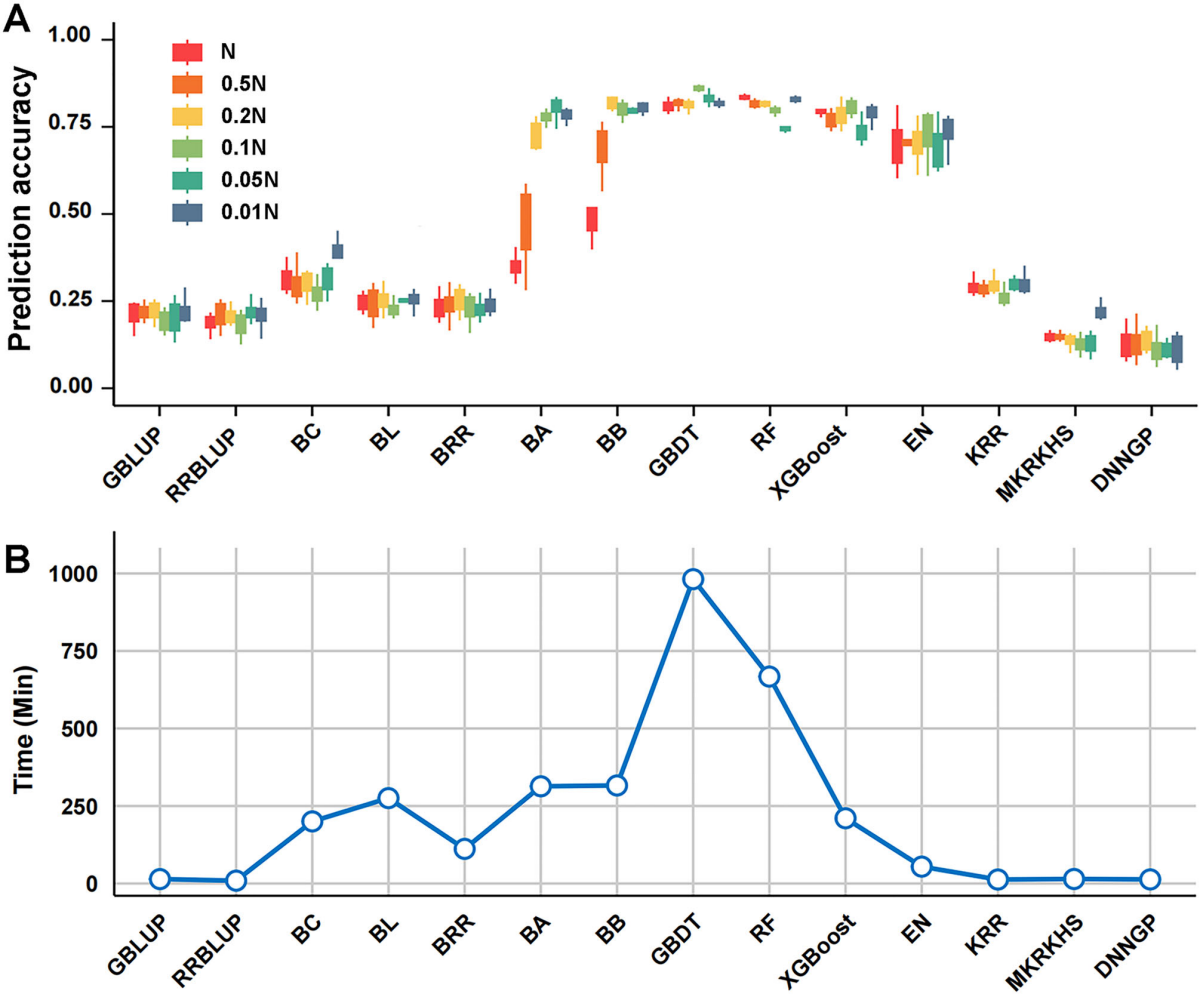
Performance comparison of 14 genomic selection (GS) statistical models. (A) Model prediction accuracy at 6 random marker densities. Random markers were generated using vcflib, with numbers ranging from 11,480 to 1,148,074. (B) Model prediction time statistics. The prediction time refers to the time required for prediction using all markers. These results are for comparison purposes only, as they depend on the specifications of the bioinformatics servers. Computations were performed on a server with AMD EPYC 7763 * 2 central processing units (CPUs) (64 cores, 128 threads) and 1024 GB memory. BA, Bayesian A; BB, Bayesian B; BC, Bayesian C; BL, Bayesian LASSO; BRR, Bayesian ridge regression; DNNGP, deep neural network Gaussian process; EN, elastic net regression; GBDT, gradient boosting decision tree; GBLUP, genomic best linear unbiased prediction; MKRKHS, multiple kernel ridge regression based on reproducing kernel Hilbert space; KRR, kernel ridge regression; RF, random forest; RRBLUP, ridge regression best linear unbiased prediction; XGBoost, eXtreme gradient boosting.

To achieve accurate sex prediction in *P. deltoides*, we first evaluated 14 mainstream GS statistical models to identify the most suitable model for sex prediction. The evaluation results revealed significant differences in predictive performance, with prediction accuracies ranging from 0.19 to 0.79 (Figure 3). There were significant differences in the predictive ability of sex across different models. Based on the evaluation results across six different marker densities, models such as BA, BB, RF, GBDT, XGBoost, and EN exhibited better predictive performance, while models like BLUP struggled to achieve high prediction accuracy (PA). Among the tested models, the GBDT model required the longest computational time, followed by RF (Figure 2B). Given that GBDT consistently demonstrated the best predictive performance across all marker densities, it was selected for subsequent analyses.

### 3.5 Impact of GWAS‐based marker selection on prediction accuracy in sex

**FIGURE 4:**
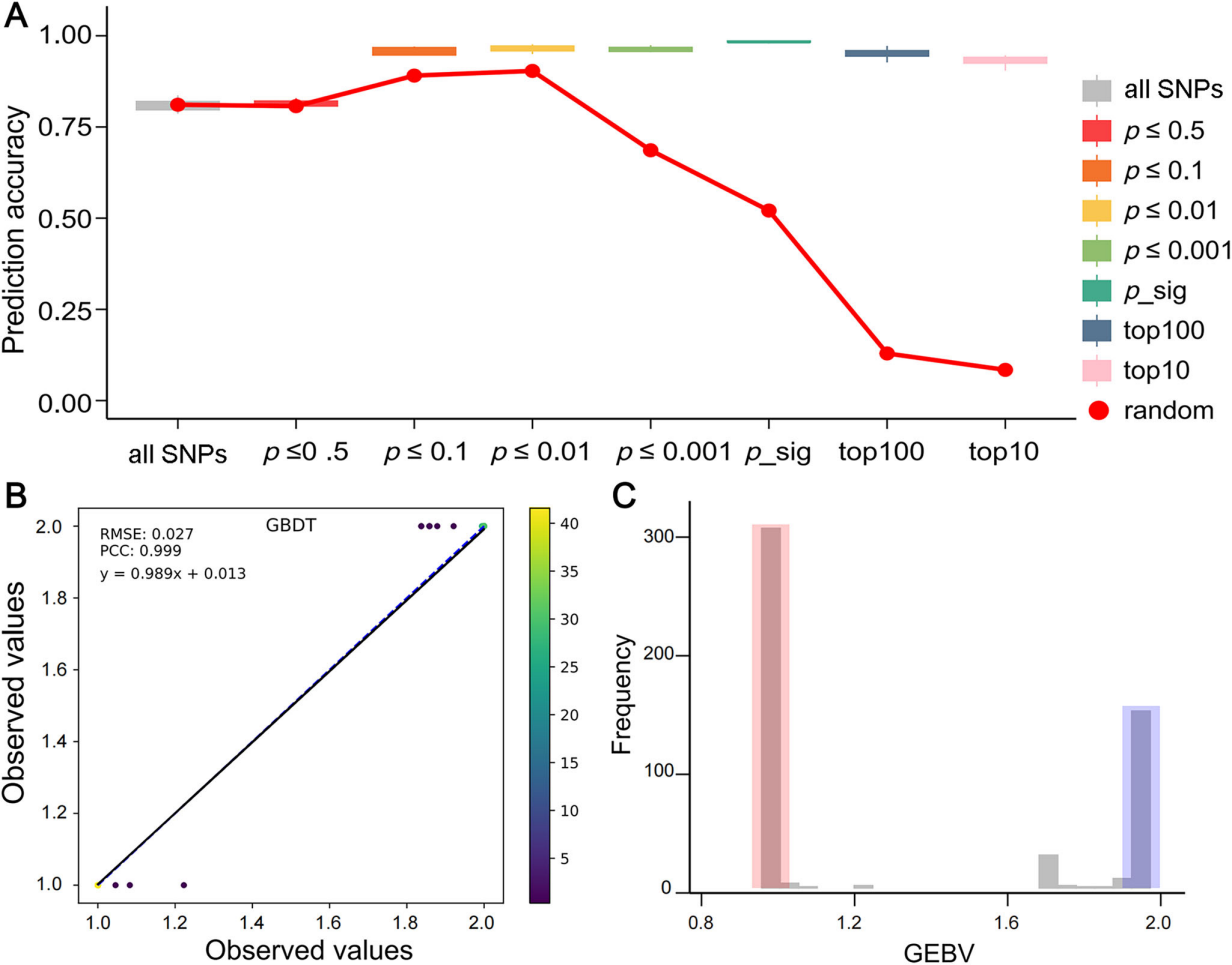
Impact of marker–trait associations on prediction accuracy (PA) and the final sex prediction model. (A) Model prediction accuracy under different marker–sex associations, based on genome‐wide association study (GWAS) *p*‐values. “all SNPs” refers to markers with *p* ≤ 1; “*p*_sig” refers to significant marks. (B) The final sex prediction model, with a prediction accuracy of 0.999, and the best‐fit equation *y* = 0.989*x* + 0.013; PCC, Pearson correlation coefficient; RMSE, root mean square error. (C) The distribution of genomic estimated breeding value (GEBV) in the breeding population, with the majority falling within the 5% confidence interval.

After evaluating the statistical model, we generated eight subsets of markers under different *p*‐value thresholds based on the GWAS association and used an equal number of random markers as controls to investigate the impact of marker–trait associations on PA and identify the optimal markers. The results showed that the model PA initially increased, then decreased as the strength of the marker–trait association increased (Figure 4A). In contrast, the PA for random markers remained relatively stable at higher densities but declined significantly as the number of markers drastically decreased. The model PA of the markers selected through GWAS screening was significantly higher than that of random markers. When using all significant markers, the model achieved the best predictive performance. Ultimately, a sex prediction model with a PA of 0.999 was established, and the linear relationship is represented as *y* = 0.989*x* + 0.013 (Figure 4B). These results demonstrate the feasibility of enhancing model PA through GWAS‐assisted marker selection.

### 3.6 Genomic prediction of breeding population

Based on the established sex prediction model, we extracted marker information from the breeding population to predict GEBV (Figure 4C). The prediction results were adjusted using a 5% confidence interval. Individuals with GEBV values between 0.95 and 1.05 were classified as female (*n* = 307), while those between 1.95 and 2.05 as male (*n* = 151). These individuals, falling within the confidence interval, were considered accurately predicted, accounting for 90.69% of the total individuals. The remaining 47 individuals, whose GEBV values fell outside the confidence interval, were deemed inaccurately predicted, potentially due to the relatively distant genetic relationship between their hybrid parents and the reference population. By integrating these accurately predicted individuals with genotype and sex data from the reference population, we expanded the cohort and conducted a follow‐up GWAS analysis. This resulted in enhanced signal strength at SDR loci (Figure S2), although it may have also increased the risk of false positives and non‐SDR regions associations. Nonetheless, these findings offer valuable insights for future GWAS research.

## 4 DISCUSSION

### 4.1 Poplar early sex prediction enhances breeding efficiency

The development of male poplar varieties has become a major focus in poplar breeding programs, as female trees produce large quantities of catkins upon reaching maturity, leading to significant environmental concerns (Ortega et al., 2023). Furthermore, our findings align with previous studies, demonstrating that male *P. deltoides* exhibit advantages in radial growth compared to the females, while no significant differences are observed in longitudinal growth (Chen et al., 2023). These sex differences further highlight the importance of early sex identification in poplar breeding, particularly given the poplar's long juvenile phase, which typically lasts 6–7 years before flowering.

Early sex GS prediction in poplar can significantly improve breeding efficiency and reduce resource waste; it also accelerates the development of male cultivars and helps mitigate the environmental pollution caused by female catkins. Currently, molecular marker identification is the primary method for early sex identification in poplar (Kim et al., 2021; Pakull et al., 2015; Wang et al., 2023). While PCR‐based markers can reliably identify male individuals, they have notable limitations. In cases where PCR amplification fails, due to low primer specificity or poor DNA quality, the sex of the individual remains uncertain and cannot be simply assigned as female. This ambiguity introduces a potential source of error in sex identification and calls for caution when interpreting non‐amplified results. Recent advancements in sequencing technologies and bioinformatics have provided new opportunities to improve the accuracy of sex determination in poplar (Gladysh et al., 2024). In this study, we present a novel method for poplar sex determination that integrates GWAS and GS to enable high‐precision sex prediction in poplar trees. To the best of our knowledge, this is also the first study in forest tree research to predict sex using GS.

### 4.2 Identification of the sex‐determining region in poplar

The accurate identification of SLRs is essential for efficient genome prediction and understanding sexual determination mechanisms. Three main methods are typically used to detect SLRs: molecular marker, sequence mapping, and GWAS, with GWAS being the most precise (Li et al., 2023). To date, the SDR of several poplar species has been successfully identified through GWAS (Gladysh et al., 2024; Müller et al., 2020; Xue et al., 2020; Zhang et al., 2022). Previous studies have demonstrated that both female and male reference genomes can localize the SDR, with highly consistent positions in both sexes (Xue et al., 2020; Zhang et al., 2022). In this study, the SDR identified in the hybrid population of *P. deltoides* coincides with the SDR location identified by Xue et al. (2020), further confirming the accuracy of the identified markers.

It is likely that multiple genes are involved in the sex determination of *Populus* species during their evolutionary process (Geraldes et al., 2015; Leite Montalvão et al., 2022; Lu et al., 2023), including genes such as *CLC‐C*, *TCP‐1*, and *MET1*. However, it is currently widely believed that only the orthologs of ARR16/ARR17 genes play a decisive role in sex determination (Müller et al., 2020). Within the SDR region, in addition to the two previously reported genes, *CLC‐C* and *MET1*, we also identified a highly conserved gene, *clptm1*, associated with Cleft lip and palate transmembrane protein 1, which may play a potential role in the sexual evolution of *P. deltoides*. These findings support the use of female reference genome for SDR identification, particularly in hybrid populations with closely related genetic relationships. However, a key limitation of using the female genome is its inability to capture Y chromosome‐specific sequences, which hinders the identification of male‐specific genes.

### 4.3 Sex prediction statistical models and performance comparison

The selection of GS models is paramount for the accuracy of genomic predictions. Previous studies have demonstrated marked significant differences in the predictive abilities of various models for traits, primarily due to differences in the assumptions and distributions of marker effects in statistical models (Momen et al., 2018). Consistent with these findings, our model evaluation revealed that among the three types of GS models, BLUP‐based methods exhibited lower predictive ability, while the Bayesian methods showed limited fitting capacity for high‐dimensional data, which may result in underfitting. Moreover, machine learning methods displayed considerable variation in predictive performance, likely due to differences in their ensemble learning architectures. Among these, both GBDT and RF demonstrated superior predictive performance, with GBDT performing the best overall.

While PA is the primary criterion for selecting GS statistical models, computational efficiency is also a critical reference factor. In this study, the computing times of different models varied significantly, in line with previous findings. In this study, we observed substantial differences in computing time among the various models, consistent with previous reports. Furthermore, our results indicated that the choice among fivefold, 10‐fold, and leave‐one‐out cross‐validation strategies had no significant impact on PA. However, these cross‐validation methods differed greatly in computational time (Figure S3), with fivefold cross‐validation offering a clear practical advantage. These findings further underscore the importance of model evaluation for different datasets and provide reference into balancing predictive performance with computational efficiency.

Currently, while GS has been widely applied in animal and plant breeding, its application in forestry tree research is still in the early stages of development (Zhu & Huang, 2020). In forestry trees, the traits of primary interest are mostly quantitative traits with complex genetic structures, influenced by various factors such as genetics, age, and environment (Resende et al., 2012). Consequently, these traits PA is generally low, typically ranging from 0.2 to 0.7 (Lebedev et al., 2020; Papin et al., 2024; Resende et al., 2012). In contrast, sex as a qualitative trait, is less influenced by environmental factors and is generally easier to predict than quantitative traits. Through comprehensive evaluation of multiple models and optimization of marker selection strategies, we achieved high‐precision prediction of sex in *P. deltoides*, with optimized PA reaching 0.999.

### 4.4 Effects of inter‐marker LD and marker–trait correlations on GP

It is well established that the accuracy of GS models is highly dependent on familial relationships. In this study, we used a multi‐generational hybrid population in which most individuals exhibited high genomic relatedness (Figure S4), thereby ensuring efficient prediction in genomic selection. Compared with natural populations, multi‐generational hybrid populations not only increase the frequency of rare variants but also possess clearer pedigree relationships, which help reduce marker detection errors.

Our findings regarding the impact of marker density on prediction accuracy are consistent with previous studies: under high‐density conditions, the PA across different marker sets were similar, but overly dense markers may lead to model underfitting (Solberg et al., 2008; Wang et al., 2024); under low‐density conditions, as the marker density decreases, the PA deteriorates significantly (Norman et al., 2018). Interestingly, unlike previous studies (Alves et al., 2020), the level of LD between markers had a relatively minor impact on prediction accuracy in this study (Figure S5), likely due to the uniformly high marker density across different LD levels. It is worth noting that this study was conducted based on a multi‐generational hybrid population, and its applicability to natural populations remains unclear.

Notably, the markers identified through GWAS can significantly improve the accuracy of sex prediction, with the use of only a few significant markers (top10) achieving a high predictive performance. When applied to the breeding population, over 90% of the individuals were accurately predicted. For those that could not be predicted, it is speculated that the reason might be due to the distant genetic relationship with the reference population, and the small number of individuals may be due to genotype imputation errors (Werner et al., 2020).

## 5 CONCLUSION

Overall, for studies planning to implement GS program, early sex prediction through GS is highly feasible. However, for studies without an existing population foundation, the genotyping cost for sex prediction through GS is very high. This study accurately identified sex‐related markers through GWAS and confirmed that using only significant markers or a small number of key markers can achieve high prediction accuracy, thereby significantly reducing the marker density for sex prediction and genotyping costs. Overall, conducting GS based on GWAS represents promise as a new approach for early sex screening in poplar trees, and it also provides valuable insights for GS research on other traits.

## AUTHOR CONTRIBUTIONS

**Xinglu Zhou**: Data curation; formal analysis; investigation; methodology; writing—original draft. **Min Zhang**: Formal analysis; investigation. **Lei Zhang**: Data curation; formal analysis; investigation; methodology; writing—original draft. **Jianjun Hu**: Funding acquisition; methodology; project administration; supervision; writing—review and editing.

## CONFLICT OF INTEREST STATEMENT

The authors declare no conflicts of interest.

## Supporting information




**Supplemental Figure S1**. QQ plot of the genome‐wide association study (GWAS) for sex.
**Supplemental Figure S2**. Manhattan plot of GWAS for sex based on accurately predicted and reference individuals.
**Supplemental Figure S3**. Prediction accuracy and computation time of the optimal statistical model for sex under different cross‐validation methods.
**Supplemental Figure S4**. Kinship heatmap of the multi‐generational hybrid population.
**Supplemental Figure S5**. The trend of model prediction accuracy at different LD levels.

## Data Availability

The whole‐genome resequencing data have been deposited under BioProject PRJCA033212. All other data related to this study are provided in the Supporting Information of the article.
