# Supplementary material for: GWAS‐assisted genomic selection for achieving high‐precision early sex prediction in Populus deltoides
Source: Plant Genome. 2026 Jan 16;19(1):e70175. doi: 10.1002/tpg2.70175 (PMC12823031; doi:10.1002/tpg2.70175)
Supplement: Supplementary file 1 [file TPG2-19-e70175-s001.docx]

**Supplemental Figure S1.** QQ plot of the genome-wide association study (GWAS) for sex.

**Supplemental Figure S2.** Manhattan plot of GWAS for sex based on accurately predicted and reference individuals.

**Supplemental Figure S3.** Prediction accuracy and computation time of the optimal statistical model for sex under different cross-validation methods.

**Supplemental Figure S4.** Kinship heatmap of the multi-generational hybrid population.

**Supplemental Figure S5.** The trend of model prediction accuracy at different LD levels.
